# Supplementary material for: Quality of life and its associated factors in chronic kidney disease patients undergoing hemodialysis from a Peruvian city: A cross-sectional study
Source: PLoS One. 2024 May 2;19(5):e0300280. doi: 10.1371/journal.pone.0300280 (PMC11065205; doi:10.1371/journal.pone.0300280)
Supplement: S1 File — (PDF) [file pone.0300280.s001.pdf]

S1 File. Data collection form

1. **Numero de historia clínica:**
2. **Características sociodemográficas:**
   - Establecimiento de salud
     - (1) Clínica del Riñón
     - (2) Seguro Social de Salud
   - Edad:
   - Género:
     - (0) Mujer
     - (1) Varón
   - Estado civil:
     - (1) Sin pareja
     - (2) Con pareja
   - Grado de instrucción:
     - (1) Sin estudios
     - (2) Con estudios escolares
     - (3) Estudio superior técnico o universitario
   - Situación laboral
     - (0) No labora
     - (1) Si labora
   - Sustento económico
     - (0) No autosustenta gastos
     - (1) Si autosustenta gastos
   - Lugar de residencia:
     - (0) Urbano
     - (1) Rural
3. **Características clínicas (presencia de comorbilidades):**
   - HTA
     - (0) No
     - (1) Si
   - DM tipo 2
     - (0) No
     - (1) Si
   - Enfermedad ateroesclerótica
     - (0) No
     - (1) Si
   - Insuficiencia cardiaca
     - (0) No
     - (1) Si
   - Enfermedad vascular periférica
     - (0) No
     - (1) Si
   - Accidente cerebrovascular
     - (0) No
     - (1) Si
   - Cáncer
     - (0) No
     - (1) Si
   - Tuberculosis
     - (0) No
     - (1) Si

3

- Ceguera
  - (0) No
  - (1) Si
- Enfermedad autoinmune
  - (0) No
  - (1) Si

4. **Características laboratoriales:**
   - Hemoglobina
   - Hematocrito
   - Albúmina
5. **Características de tratamiento:**
   - Tratamiento administrado durante la HD
   - Frecuencia de HD a la semana
   - Tiempo de HD en años
   - Horas por cada HD
   - Tipo de acceso vascular:
     - (0) CVC
     - (1) FAV

4

5

1. En general, usted diría que su salud es:

☐ 1	☐ 2	☐ 3	☐ 4	☐ 5
Excelente	Muy buena	Buena	Regular	Mala

2. ¿Cómo diría usted que es su salud actual, comparada con la de hace un año?:

Mucho mejor ahora que hace un año	Algo mejor ahora que hace un año	Más o menos igual que hace un año	Algo peor ahora que hace un año	Mucho peor ahora que hace un año
☐ 1	☐ 2	☐ 3	☐ 4	☐ 5

3. Las siguientes preguntas se refieren a actividades o cosas que usted podría hacer en un día normal. Su salud actual, ¿le limita para hacer esas actividades o cosas? Si es así, ¿cuánto?

	Sí, me limita mucho	Sí, me limita un poco	No, no me limita nada
a **Esfuerzos intensos**, tales como correr, levantar objetos pesados, o participar en deportes agotadores.	☐ 1	☐ 2	☐ 3
b **Esfuerzos moderados**, como mover una mesa, pasar la aspiradora, jugar a los bolos o caminar más de 1 hora.	☐ 1	☐ 2	☐ 3
c Coger o llevar la bolsa de la compra.	☐ 1	☐ 2	☐ 3
d Subir varios pisos por la escalera.	☐ 1	☐ 2	☐ 3
e Subir un sólo piso por la escalera.	☐ 1	☐ 2	☐ 3
f Agacharse o arrodillarse.	☐ 1	☐ 2	☐ 3
g Caminar un kilómetro o más	☐ 1	☐ 2	☐ 3
h Caminar varios centenares de metros.	☐ 1	☐ 2	☐ 3
i Caminar unos 100 metros.	☐ 1	☐ 2	☐ 3
j Bañarse o vestirse por sí mismo.	☐ 1	☐ 2	☐ 3

4. Durante las 4 últimas semanas, ¿con qué frecuencia ha tenido alguno de los siguientes problemas en su trabajo o en sus actividades cotidianas, a causa de su salud física?

	Siempre	Casi siempre	Algunas veces	Sólo alguna vez	Nunca
a ¿Tuvo que reducir el tiempo dedicado al trabajo o a sus actividades cotidianas?	☐ 1	☐ 2	☐ 3	☐ 4	☐ 5
b ¿Hizo menos de lo que hubiera querido hacer?	☐ 1	☐ 2	☐ 3	☐ 4	☐ 5
c ¿Tuvo que dejar de hacer algunas tareas en su trabajo o en sus actividades cotidianas?	☐ 1	☐ 2	☐ 3	☐ 4	☐ 5
d ¿Tuvo dificultad para hacer su trabajo o sus actividades cotidianas (por ejemplo, le costó más de lo normal)?	☐ 1	☐ 2	☐ 3	☐ 4	☐ 5

5. Durante las 4 últimas semanas, ¿con qué frecuencia ha tenido alguno de los siguientes problemas en su trabajo o en sus actividades cotidianas, a causa de algún problema emocional (como estar triste, deprimido o nervioso)?

	Siempre	Casi siempre	Algunas veces	Sólo alguna vez	Nunca
a ¿Tuvo que reducir el tiempo dedicado al trabajo o a sus actividades cotidianas por algún problema emocional?	☐ 1	☐ 2	☐ 3	☐ 4	☐ 5
b ¿Hizo menos de lo que hubiera querido hacer por algún problema emocional?	☐ 1	☐ 2	☐ 3	☐ 4	☐ 5
c ¿Hizo su trabajo o sus actividades cotidianas menos cuidadosamente que de costumbre, por algún problema emocional?	☐ 1	☐ 2	☐ 3	☐ 4	☐ 5

6. Durante las 4 últimas semanas, ¿hasta qué punto su salud física o los problemas emocionales han dificultado sus actividades sociales habituales con la familia, los amigos, los vecinos u otras personas?

Nada	Un poco	Regular	Bastante	Mucho
☐ 1	☐ 2	☐ 3	☐ 4	☐ 5

7. ¿Tuvo dolor en alguna parte del cuerpo durante las 4 últimas semanas?

No, ninguno	Sí, muy poco	Sí, un poco	Sí, moderado	Sí, mucho	Sí, muchísimo
☐ 1	☐ 2	☐ 3	☐ 4	☐ 5	☐ 6

8. Durante las 4 últimas semanas, ¿hasta qué punto el dolor le ha dificultado su trabajo habitual (incluido el trabajo fuera de casa y las tareas domésticas)?

Nada	Un poco	Regular	Bastante	Mucho
☐ 1	☐ 2	☐ 3	☐ 4	☐ 5

9. Las preguntas que siguen se refieren a cómo se ha sentido y cómo le han ido las cosas durante las 4 últimas semanas. En cada pregunta responda lo que se parezca más a cómo se ha sentido usted. Durante las últimas 4 semanas ¿con qué frecuencia...

	Siempre	Casi siempre	Algunas veces	Sólo alguna vez	Nunca
a se sintió lleno de vitalidad?	☐ 1	☐ 2	☐ 3	☐ 4	☐ 5
b estuvo muy nervioso?	☐ 1	☐ 2	☐ 3	☐ 4	☐ 5
c se sintió tan bajo de moral que nada podía animarle?	☐ 1	☐ 2	☐ 3	☐ 4	☐ 5
d se sintió calmado y tranquilo?	☐ 1	☐ 2	☐ 3	☐ 4	☐ 5
e tuvo mucha energía?	☐ 1	☐ 2	☐ 3	☐ 4	☐ 5
f se sintió desanimado y deprimido?	☐ 1	☐ 2	☐ 3	☐ 4	☐ 5
g se sintió agotado?	☐ 1	☐ 2	☐ 3	☐ 4	☐ 5
h se sintió feliz?	☐ 1	☐ 2	☐ 3	☐ 4	☐ 5
i se sintió cansado?	☐ 1	☐ 2	☐ 3	☐ 4	☐ 5

10. Durante las 4 últimas semanas, ¿con qué frecuencia la salud física o los problemas emocionales le han dificultado sus actividades sociales (como visitar a los amigos o familiares)?

Siempre	Casi siempre	Algunas veces	Sólo alguna vez	Nunca
☐ 1	☐ 2	☐ 3	☐ 4	☐ 5

11. Por favor diga si le parece CIERTA o FALSA cada una de las siguientes frases:

	Totalmente cierta	Bastante cierta	No lo sé	Bastante falsa	Totalmente falsa
a Creo que me pongo enfermo más facilmente que otras personas	☐ 1	☐ 2	☐ 3	☐ 4	☐ 5
b Estoy tan sano como cualquiera	☐ 1	☐ 2	☐ 3	☐ 4	☐ 5
c Creo que mi salud va a empeorar	☐ 1	☐ 2	☐ 3	☐ 4	☐ 5
d Mi salud es excelente	☐ 1	☐ 2	☐ 3	☐ 4	☐ 5

CUESTIONARIO HSCL-25

	Síntomas de Ansiedad	(1) Nada	(2) Un poco	(3) Bastante	(4) Mucho
1	Se asusta súbitamente sin motivo				
2	Siente que tiene miedo				
3	Se desmaya, se marea o se siente débil				
4	Siente nerviosismo o inquietud en su interior				
5	Su corazón late aceleradamente o más rápido de lo usual				
6	Temblor				
7	Se siente tenso/a o entrampado/a				
8	Dolores de cabeza				
9	Períodos de terror o pánico				
10	Siente inquietud o que no puede estar tranquilo				

	Síntomas de Depresión	(1) Nada	(2) Un poco	(3) Bastante	(4) Mucho
11	Siente falta de fuerzas, lentitud				
12	Tiene sentimiento de culpa persistente. Remordimientos				
13	Llora fácilmente				
14	Ha perdido el interés sexual o placer				
15	Falta de apetito				
16	Dificultad para dormir o continuar dormido				
17	Se siente desesperanzado/a sobre el futuro				
18	Se siente triste				
19	Se siente solo/a				
20	Ha pensado acabar con su vida				
21	Se siente atrapado o aprisionado				
22	Se preocupa excesivamente por las cosas				
23	Siente falta de interés por las cosas				
24	Siente que todo requiere demasiado esfuerzo				
25	Se siente inútil				
