# Supplementary material for: Quality of life and its associated factors in chronic kidney disease patients undergoing hemodialysis from a Peruvian city: A cross-sectional study
Source: PLoS One. 2024 May 2;19(5):e0300280. doi: 10.1371/journal.pone.0300280 (PMC11065205; doi:10.1371/journal.pone.0300280)
Supplement: Attachment — Submitted filename: Response letter.docx [file pone.0300280.s003.docx]

Dear reviewers and editor, in this letter we respond to your observations:

Editor's Comments:

1. Please ensure that your manuscript meets PLOS ONE's style requirements, including those for file naming.
   a. It was made according to PLOS ONE's style requirements.
2. Please provide additional details regarding participant consent. In the ethics statement in the Methods and online submission information, please ensure that you have specified what type you obtained (for instance, written or verbal, and if verbal, how it was documented and witnessed). If your study included minors, state whether you obtained consent from parents or guardians. If the need for consent was waived by the ethics committee, please include this information.
   a. More details were given in the ethics section. Line 145 – 147.
3. Thank you for stating the following in the Acknowledgments Section of your manuscript: "We would like to acknowledge the Scientific University of the South for its support in paying the costs of processing the article." We note that you have provided funding information that is not currently declared in your Funding Statement. However, funding information should not appear in the Acknowledgments section or other areas of your manuscript. We will only publish funding information present in the Funding Statement section of the online submission form. Please remove any funding-related text from the manuscript and let us know how you would like to update your Funding Statement. Currently, your Funding Statement reads as follows: "The author(s) received no specific funding for this work". Please include your amended statements within your cover letter; we will change the online submission form on your behalf.
   a. Dear editor, we have made the modification and we have removed the thanks and detailed the APC payment in the corresponding section.
4. All PLOS journals now require all data underlying the findings described in their manuscript to be freely available to other researchers, either 1. In a public repository, 2. Within the manuscript itself, or 3. Uploaded as supplementary information. This policy applies to all data except where public deposition would breach compliance with the protocol approved by your research ethics board. If your data cannot be made publicly available for ethical or legal reasons (e.g., public availability would compromise patient privacy), please explain your reasons on resubmission and your exemption request will be escalated for approval.
   a. Dear editor, we attach the study database as supplementary information (S2_File) in Excel spreadsheet format; Sheet one contains the complete information that was analyzed in the study and sheet two contains the data of each variable as well as its interpretation.
5. Review your reference list to ensure that it is complete and correct. If you have cited papers that have been retracted, please include the rationale for doing so in the manuscript text, or remove these references and replace them with relevant current references. Any changes to the reference list should be mentioned in the rebuttal letter that accompanies your revised manuscript. If you need to cite a retracted article, indicate the article’s retracted status in the References list and also include a citation and full reference for the retraction notice.
   a. We have carried out the corresponding review.

Revisión:

6. Reviewer #1: Line 162, only 53.1% had 3 hours session; what about the other 46.9%? Did they have less or more than 3 hours of session? It may be interesting to compare the QOL between those with a higher number of hours per month vs lower numbers of hemodialysis per month.
   a. Dear reviewer, we appreciate your observation, 46.9% were patients who had hemodialysis lasting 3 and a half hours; we added this information in the manuscript. Although this variable is interesting to analyze, it is the patients treated at EsSalud who underwent hemodialysis for 3 hours and those at the Kidney Clinic who underwent hemodialysis for 3 and a half hours. Therefore, this variable could not be included in the multivariable model.
7. Reviewer #2: The manuscript describes quality of life amongst dialysis patients in 2 hospitals in the city of Tacna, Peru. Data was collected using telephone surveys with the HrQoL 36 form. The methodology and the exclusion criteria are quite clear. The authors state that the interviewers had training sessions as follows: "Two researchers (D.M.C. and G.P.G.) underwent two training sessions in interviews and data collection, each lasting 45 minutes". They should also state the average length of the phone interviews for better understanding of the data collection process.
   a. Your observation about the length of each interview is an interesting point to analyze. But we were not able to obtain this value since we did not take the time of each interview. When conducting the consultation, the interviewers reported that on average the time of each interview was between 20 and 30 minutes. Since this data is not objective, we added it as a limitation of the study.

We consider that this new version of the manuscript has improved its quality thanks to your comments.

Kind regards

Dr. Cesar Copaja Corzo (Corresponding autor)
